# Supplementary material for: EventDTW: An Improved Dynamic Time Warping Algorithm for Aligning Biomedical Signals of Nonuniform Sampling Frequencies
Source: Sensors (Basel). 2020 May 9;20(9):2700. doi: 10.3390/s20092700 (PMC7273204; doi:10.3390/s20092700)
Supplement: Supplementary file 1 [file sensors-20-02700-s001.pdf]

sensors

Supplementary Materials

EventDTW: An Improved Dynamic Time Warping Algorithm for Aligning Biomedical Signals of Nonuniform Sampling Frequencies

Yihang Jiang [1,†], Yuankai Qi [1,†], Will Ke Wang [1], Brinnae Bent [1], Robert Avram [2], Jeffery Olgin [2], Jessilyn Dunn [1,*]

[1] The Departments of Biomedical Engineering and Biostatistics & Bioinformatics, Duke University, Durham, NC 27708 USA,
[2] The Division of Cardiology and the Cardiovascular Research Institute, University of California San Francisco, San Francisco, CA 94143
* Correspondence: Jessilyn.dunn@duke.edu
† First co-authors

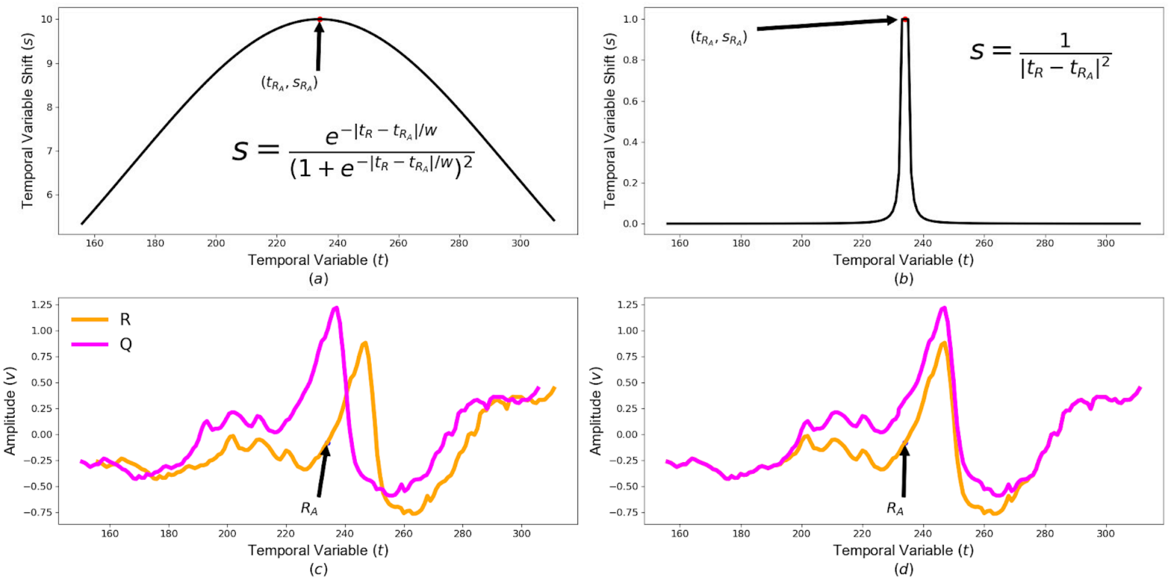

Figure S1. Comparison between temporal variable shift functions

Figure 1. (a) (b)Temporal variable shift function designed based on the derivative of sigmoid function and inverse square distance function. (c) (d) Part of signal near anchor point in Beef dataset from UCR Time Series Classification Archive warped based on (a) and (b)

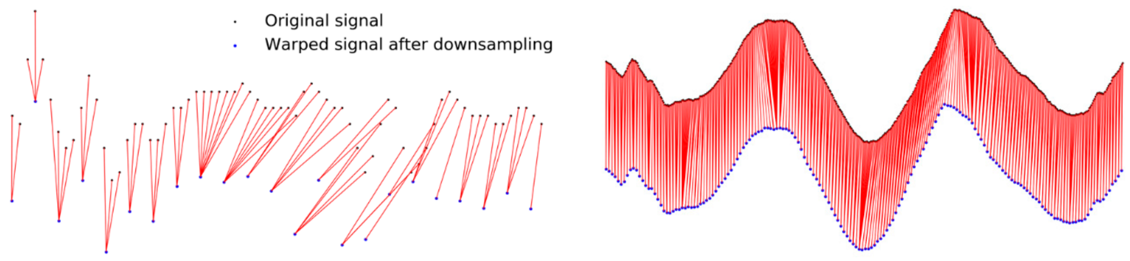

Figure S2. Examples in which eDTW perform worse than DTW.

Table S1. The Error Rate and Singularity Score for 84 datasets from the UCR Time Series Classification Archive

	Event DTW ER	Event DTW SS	DTW ER	DTW SS	shape DTW ER	shape DTW SS	dDTW ER	dDTW SS
50words_TRAIN	0.010204	5.582889	0.010882	5.86042	0.028369	13.56741	0.023171	7.586778
Adiac_TRAIN	0.004537	0.866667	0.004547	0.901149	0.071597	21.57701	0.08532	23.41825
ArrowHead_TRAIN	0.014835	11.3366	0.013953	11.11402	0.044397	20.52209	0.030228	13.20623
Beef_TRAIN	0.00879	9.790832	0.009682	11.14829	0.020516	22.33162	0.012517	8.499793
BeetleFly_TRAIN	0.002333	1.445588	0.002362	1.537941	0.019245	18.75137	0.020132	22.50025
BirdChicken_TRAIN	0.006504	4.813336	0.005525	3.907878	0.031649	40.24	0.031255	25.01438
Car_TRAIN	0.006838	5.654083	0.006723	5.026461	0.044303	83.41701	0.036464	34.14809
CBF_TRAIN	0.026674	6.291799	0.026674	6.291799	0.098568	38.93968	0.056078	8.672222
ChlorineConcentration_TRAIN	0.013165	3.965556	0.013143	3.931616	0.095589	36.77212	0.052783	7.167475
Coffee_TRAIN	0.006084	4.246942	0.005857	4.172206	0.022721	12.22526	0.016917	7.493684
Computers_TRAIN	0.135313	267.082	0.135313	267.082	0.164272	306.2588	0.132968	256.4508
CinC_ECG_torso_TRAIN	0.004852	7.79796	0.007242	9.029677	0.056561	243.3684	0.004813	8.396662
Cricket_X_TRAIN	0.00863	4.784567	0.008586	4.772567	0.032168	18.99867	0.01902	6.622278
Cricket_Y_TRAIN	0.014377	6.838956	0.014199	6.737623	0.035916	21.348	0.018684	7.438389
Cricket_Z_TRAIN	0.016024	5.644553	0.01597	5.660553	0.045566	32.701	0.019071	7.252578
DiatomSizeReduction_TRAIN	0.006954	3.90456	0.004073	0.995623	0.072693	65.67072	0.077625	35.92303
DistalPhalanxOutlineAgeGroup_TRAIN	0.012205	3.425641	0.010513	3.487179	0.072923	7.112821	0.050256	5.979487
DistalPhalanxOutlineCorrect_TRAIN	0.013949	3.805128	0.011487	3.911538	0.07441	7.266667	0.049077	5.911538

DistalPhalanxTW_TRAIN	0.043333	7.728205	0.041846	7.728205	0.094615	8.523077	0.070667	10.80855
Earthquakes_TRAIN	0.09073	62.45509	0.09073	62.45509	0.196842	139.0679	0.130171	95.34793
ECG200_TRAIN	0.018918	3.534375	0.019153	3.580208	0.053394	11.38333	0.061492	10.41076
ECG5000_TRAIN	0.018164	4.437246	0.017681	4.222754	0.052448	15.7971	0.047746	15.54614
ECGFiveDays_TRAIN	0.012542	3.826667	0.012727	3.900741	0.03867	10.96963	0.033333	7.96321
ElectricDevices_TRAIN	0.317036	93.39444	0.317036	93.39444	0.238844	70.60833	0.449227	126.5423
FaceAll_TRAIN	0.01495	2.659948	0.013972	2.595866	0.099077	30.02171	0.036656	3.734884
FaceFour_TRAIN	0.010335	6.777783	0.010335	6.777783	0.016309	8.981322	0.011449	5.137644
FacesUCR_TRAIN	0.011831	3.525194	0.011886	3.562403	0.082097	18.95426	0.02942	3.810853
FISH_TRAIN	0.01046	8.76279	0.009443	8.022411	0.052624	63.72424	0.057795	52.70938
FordA_TRAIN	0.001969	1.728514	0.001976	1.75502	0.011184	7.861847	0.006685	3.274096
FordB_TRAIN	0.001958	1.740228	0.001978	1.775569	0.009652	5.722088	0.006557	3.044177
Gun_Point_TRAIN	0.022748	7.518111	0.018054	6.551444	0.057224	20.06667	0.058286	15.10686
Ham_TRAIN	0.006154	5.223997	0.006226	5.385069	0.011281	7.773427	0.007799	3.451282
HandOutlines_TRAIN	0.003292	3.820319	0.002961	1.249506	0.170416	1658.368	0.047254	204.0377
Haptics_TRAIN	0.005766	9.202662	0.005596	9.573793	0.022467	94.0207	0.013308	21.43163
Herring_TRAIN	0.005874	4.737265	0.006563	4.756971	0.046537	62.71059	0.027619	27.26876
InlineSkate_TRAIN	0.005891	21.97075	0.005884	21.96285	0.112127	641.4684	0.008277	11.42907
InsectWingbeatSound_TRAIN	0.018497	8.359625	0.024949	9.783654	0.030724	11.04588	0.023604	7.670211
ItalyPowerDemand_TRAIN	0.0625	5.445833	0.0625	5.445833	0.181548	8.55	0.152381	5.984722
LargeKitchenAppliances_TRAIN	0.225683	421.717	0.238333	434.0703	0.222641	483.9351	0.190877	364.6064

Lighting2_TRAIN	0.039389	34.13364	0.039383	34.12924	0.063774	85.22951	0.022598	26.93007
Lighting7_TRAIN	0.037781	24.33291	0.037799	24.2675	0.086143	58.45122	0.038263	19.22487
MALLAT_TRAIN	0.006318	16.27145	0.007074	17.35355	0.011237	29.38128	0.009914	12.21429
Meat_TRAIN	0.009933	9.377442	0.02393	24.43881	0.038529	44.20865	0.015473	7.028596
MedicalImages_TRAIN	0.049684	6.122795	0.049874	6.159158	0.097159	15.39125	0.111269	8.955387
MiddlePhalanxOutlineAgeGroup_TRAIN	0.015744	3.861538	0.011333	3.788462	0.063077	6.697436	0.055949	6.6
MiddlePhalanxOutlineCorrect_TRAIN	0.015538	3.670513	0.011692	3.648718	0.060308	6.574359	0.056718	6.292308
MiddlePhalanxTW_TRAIN	0.051436	8.707692	0.046821	8.574359	0.080667	8.220513	0.084051	12.77949
MoteStrain_TRAIN	0.018078	3.69246	0.017549	3.625794	0.087919	10.27381	0.077954	12.4627
NonInvasiveFatalECG_Thorax1_TRAIN	0.024154	31.58702	0.024419	31.36622	0.023702	48.17387	0.010018	8.795235
NonInvasiveFatalECG_Thorax2_TRAIN	0.019792	34.78682	0.025406	36.96413	0.031171	60.85468	0.01243	10.3133
OliveOil_TRAIN	0.011179	11.4396	0.011111	11.41995	0.01617	20.39824	0.008658	5.795497
OSULeaf_TRAIN	0.003408	2.005908	0.00341	2.024276	0.022615	21.6108	0.020507	18.84976
PhalangesOutlinesCorrect_TRAIN	0.015641	3.547436	0.012308	3.557692	0.062769	7.123077	0.053795	6.526923
Phoneme_TRAIN	0.001929	2.899617	0.001919	2.871204	0.064261	71.37175	0.004492	3.61247
Plane_TRAIN	0.006531	2.171528	0.006413	2.149306	0.042405	10.92222	0.039303	10.05185
ProximalPhalanxOutlineAgeGroup_TRAIN	0.011795	2.769231	0.010821	2.974359	0.06359	5.938462	0.054769	7.938462
ProximalPhalanxOutlineCorrect_TRAIN	0.011385	2.620513	0.010154	2.815385	0.06441	5.871795	0.054103	7.779487
ProximalPhalanxTW_TRAIN	0.05841	9.548718	0.056718	9.574359	0.085897	8.05641	0.089385	15.18291
RefrigerationDevices_TRAIN	0.015983	33.3118	0.015983	33.31846	0.00975	14.7925	0.012315	29.13746
ScreenType_TRAIN	0.097535	181.8684	0.097535	181.8684	0.1233	250.1428	0.088251	153.2955

ShapeletSim_TRAIN	0.008799	6.584806	0.008783	6.573561	0.295809	474.6851	0.03595	10.95385
ShapesAll_TRAIN	0.005661	2.547353	0.0052	1.976046	0.045394	55.75608	0.063887	64.40488
SmallKitchenAppliances_TRAIN	0.120223	258.4807	0.120223	258.4807	0.093927	192.3871	0.106317	227.2055
SonyAIBORobotSurfaceII_TRAIN	0.036984	5.37619	0.036984	5.37619	0.117778	14.08095	0.117778	10.2582
SonyAIBORobotSurface_TRAIN	0.02253	5.937681	0.022134	5.676812	0.076943	7.997101	0.067984	7.894203
StarLightCurves_TRAIN	0.014578	14.89606	0.01522	14.14699	0.044139	91.66374	0.017614	20.922
Strawberry_TRAIN	0.011383	5.561068	0.011244	5.631154	0.023959	12.54786	0.016311	6.247721
SwedishLeaf_TRAIN	0.006911	1.746032	0.006059	1.49127	0.048161	13.9746	0.051878	13.52553
Symbols_TRAIN	0.016987	7.720507	0.017791	9.298748	0.075914	100.0572	0.046397	34.80104
synthetic_control_TRAIN	0.062544	7.28	0.062544	7.28	0.259649	46.91333	0.138158	8.677778
ToeSegmentation1_TRAIN	0.010854	5.833979	0.010547	5.64974	0.021385	9.823913	0.015823	5.723896
ToeSegmentation2_TRAIN	0.009874	6.192324	0.009789	6.28365	0.017166	12.02398	0.013551	6.34654
Trace_TRAIN	0.041819	18.0756	0.043105	18.35202	0.10361	76.22857	0.037106	13.45072
TwoLeadECG_TRAIN	0.010494	3.024691	0.010446	3.054321	0.051709	9.553086	0.052754	9.432099
Two_Patterns_TRAIN	0.027952	8.698677	0.027952	8.698677	0.066686	19.56429	0.042741	10.46111
UWaveGestureLibraryAll_TRAIN	0.008734	18.78447	0.008785	18.91981	0.009982	19.46286	0.009238	19.35495
uWaveGestureLibrary_X_TRAIN	0.022308	15.39384	0.023526	16.17375	0.037131	28.42609	0.033404	21.95961
uWaveGestureLibrary_Y_TRAIN	0.026679	19.49531	0.028178	20.89332	0.03631	25.03905	0.030916	22.38201
uWaveGestureLibrary_Z_TRAIN	0.023452	16.7886	0.023245	16.94094	0.040449	28.32222	0.030351	20.46337
wafer_TRAIN	0.067374	21.93005	0.067442	21.74761	0.09083	22.91477	0.075524	23.94341
Wine_TRAIN	0.019814	9.892557	0.019825	9.91307	0.027733	9.528205	0.053979	26.71275

WordsSynonyms_TRAIN	0.010995	4.872093	0.010162	4.893113	0.02598	15.70481	0.024382	10.5263
WormsTwoClass_TRAIN	0.004894	7.085366	0.004828	7.002669	0.009813	18.74133	0.006631	10.73929

Table S2. The Singularity Score for 38 pairs of signals from the biomedical dataset STEP

EventDTW SS	DTW SS	shapeDTW SS	dDTW SS
214.7496	255.9367	6507.57	916.4238
652.0578	706.6031	9763.598	674.9932
207.9946	248.1262	8267.112	585.3234
863.2967	931.8183	9867.111	1840.99
271.7775	307.3894	6588.195	1014.131
312.8843	365.8864	8321.331	1420.998
199.4667	236.4396	5508.966	326.0338
165.706	202.4154	6240.588	521.357
151.0617	174.6621	3823.265	379.7549
726.1928	799.8118	9644.092	764.3193
98.12004	133.1142	5389.097	544.5346
234.3362	280.7707	6500.829	796.0459
210.5184	227.6504	2586.921	357.2649
407.1318	437.6987	5318.644	316.0044
596.9201	631.4459	5006.234	397.1649
827.2896	872.0031	8137.852	752.6112
338.6973	373.6216	5413.265	447.3042
360.1413	384.7664	4580.263	243.7712
567.2219	613.0839	7246.203	486.145
435.1125	458.8609	3372.282	254.1326
331.0873	362.1576	5033.389	527.209
419.9987	470.5904	6728.692	899.2313
476.1483	513.0718	6276.994	670.0777
166.6122	219.0746	9495.696	845.2175
462.5991	505.0618	6496.791	1006.34
179.9846	194.3681	2704.09	99.84207
265.9964	305.0589	6562.5	382.5716
453.4637	492.3472	6104.72	719.726
243.463	267.893	3835.516	214.2897
306.9594	339.4027	5547.813	443.2662
382.8466	411.8045	4633.256	709.9841
171.4716	210.6143	6106.256	1021.483
280.4449	334.315	8565.34	1669.624
1115.824	1161.347	7738.876	1273.979
266.8022	326.4465	8827.358	1065.5

202.3897	232.9697	3577.855	222.8987
740.5745	778.6155	5896.361	273.463
275.5137	310.9087	5592.405	453.0661

© 2020 by the authors. Licensee MDPI, Basel, Switzerland. This article is an open access article distributed under the terms and conditions of the Creative Commons Attribution (CC BY) license (http://creativecommons.org/licenses/by/4.0/).
